# Diabetes mellitus and endometrial carcinoma: Risk factors and etiological links

**DOI:** 10.1097/MD.0000000000030299

**Published:** 2022-08-26

**Authors:** Ya Wang, Xinling Zeng, Jie Tan, Yi Xu, Cunjian Yi

**Affiliations:** a Department of Endocrinology, The First Affiliated Hospital of Yangtze University, Jingzhou First People’s Hospital, Jingzhou, Hubei, China; b Department of Clinical Medical Research Center for Personalized Diagnosis and Treatment of Cancer, The First Affiliated Hospital of Yangtze University, Jingzhou First People’s Hospital, Jingzhou, Hubei, China; c Department of gynaecology and obstetrics,The First School of Clinical Medicine,Yangtze University, Jingzhou, Hubei, China; d Department of Hematology, The First Affiliated Hospital of Yangtze University, Jingzhou First People’s Hospital, Jingzhou, Hubei, China.

**Keywords:** diabetes mellitus, endometrial cancer, etiological links, hyperglycemia

## Abstract

The presence of diabetes mellitus (DM) has a critical influence on the occurrence and development of endometrial cancer (EC) and is associated with a poor prognosis. Patients with DM are twice as likely to progress to EC, probably because a high-glucose environment contributes to the growth and invasiveness of EC cells. In this review, we focus on the etiological links between DM and EC and provide an overview of potential biological mechanisms that may account for this relationship, including hyperglycemia, insulin resistance, hyperinsulinemia, glycolysis, chronic inflammation, obesity, and activation of signaling pathways involved in EC. Furthermore, we discuss the pharmacological management of EC associated with DM. Early treatment with metformin is expected to be an effective adjuvant alternative for EC in the future. This knowledge is important for further opening up preventive and therapeutic strategies for EC by targeting glucose metabolism.

## 1. Introduction

Endometrial cancer (EC) is the most prevalent gynecological malignancy that has grown rapidly at a rising rate with increased incidence and mortality worldwide in recent years. According to statistics, in 2019, there were more than 60,000 new cases and 12,000 deaths from EC, making it the fourth most common cancer in women in the United States.^[1]^ EC is a type of adenocarcinoma originating from intrauterine epithelial cells, and the surrounding stromal cells, endothelial cells and immune cells constitute the tumor microenvironment and thus stimulate the surrounding stromal cells to produce energy-rich catabolic metabolites to promote the growth and survival of cancer cells, which ultimately affects the progression of cancer and the response to treatment.^[2]^

Diabetes mellitus (DM) is a group of complex metabolic disorders characterized by hyperglycemia, insulin resistance, and hyperinsulinemia. Currently, DM has become a major cause of mortality and complications worldwide due to its increasing incidence, thus seriously affecting people’s overall quality of life.^[3]^ The high prevalence of DM especially type-2 DM (T2DM), has been accompanied by rising rates of several types of cancers, leading to the hypothesis that there may be a direct link between DM and cancers.^[4]^ Actually, T2DM is considered to be associated with an increased risk for different types of cancers including hepatocellular, pancreatic, breast, ovarian, gastrointestinal and endometrial cancers.^[5–7]^ Growing evidence has suggested that DM is a potential contributor to the higher incidence of EC and is associated with poor prognosis for this disease.^[8]^ According to epidemiologic studies, hyperglycemia is an independent risk factor for EC development, and patients with DM are twice as likely to progress to EC, probably because a high-glucose environment may contribute to the growth and invasiveness of EC cells.^[9,10]^ However, the etiological relationship between DM and EC is complex and poorly understood, and the precise biological mechanisms linking DM and EC remain unclear. Several studies suggest that it may be possible to increase the risk of EC in patients with diabetes, including common risk factors such as diet, age, obesity, smoking, lack of exercise, and metabolic abnormalities. Furthermore, although the specific mechanisms involved are not completely understood, the underlying mechanisms, including hyperglycemia, insulin resistance (IR), hyperinsulinemia, glycolysis, chronic inflammation, obesity, and activation of specific signaling pathways, may contribute to the increased risk of EC in diabetic patients. Taken together, these data suggest that DM has a critical influence on the occurrence and development of EC. Since the present treatments are still not completely valid in preventing or delaying progression to EC, exploration of the early and effective prevention and treatments by targeting glucose metabolism may provide new insights into targeted therapeutic intervention in EC, and have significant medical and social value.

In this review, we focus on the etiological relationship between DM and EC and provide an overview of the effects of DM on EC on hyperglycemia, insulin resistance, hyperinsulinemia, glycolysis, chronic inflammation, obesity, activation of signaling pathways involved, and pharmacological therapy of EC associated with DM. This knowledge is important for further opening up preventive and therapeutic strategies for EC by targeting glucose metabolism.

## 2. Epidemiologic studies linking DM and EC

Most epidemiological studies indicate that EC is strongly associated with T2DM. An analysis of T2DM and cancer risk and mortality showed that T2DM significantly increased both the morbidity and mortality of EC, with a summary RR of 1.97 (95% CI: 1.71–2.27) and 1.23 (95% CI: 0.78–1.93), respectively.^[5]^ At the same time, in addition to T2DM patients, the risk of EC has increased by 4.9% in the early stage of DM (RR: 1.60; 95% CI: 1.13–2.27).^[11]^ Also, T1DM increased the risk of EC, with a summary HR of 1.42 (95% CI: 1.27–1.58).^[12]^ In addition, Except for increased incidence of EC, DM may be an independent risk factor for EC mortality.^[13,14]^ According to the data of a previous study among Asian populations, DM had increased the risk of almost all cancer deaths by 26% (HR: 1.26; 95% CI: 1.21–1.31), of which the HR for EC was 2.73 (95% CI:1.53-4.85).^[14]^ Another study found robust observational evidence for the association between T2DM and EC, and showed that T2DM significantly increased both the incidence and mortality of EC, with a summary RR of 1.65 (95% CI: 1.50–1.81) and 1.32 (95% CI: 1.13–1.55), respectively.^[15]^ Furthermore, there was another study indicated that cancer patients with DM that were not actively controlled had a worse prognosis than those without DM.^[16]^ Taken together, these data strongly suggest that DM increases the incidence and mortality of EC (Table 1).

**Table 1:** Epidemiologic studies linking DM and EC.

Incidence of cancer or mortality	Type of DM	No. of cases	Random effects	*P* value	References
Mortality			(95% CI)		
Incidence	Prediabetes	966	1.60 (1.13–2.27)	0.008	^[11]^
	T2DM	8174	1.97 (1.71–2.27)	<0.001	^[5]^
	T2DM	16,224	1.65 (1.50–1.81)	<0.05	^[15]^
	TIDM	323	1.42 (1.27–1.58)	<0.05	^[12]^
	DM	7698	1.72 (1.48–2.01)	0.000	^[13]^
Mortality	T2DM	103	1.23 (0.78–1.93)	0.38	^[5]^
	T2DM	158	2.73 (1.53–4.85)	<0.05	^[14]^
	T2DM	2070	1.32 (1.13–1.55)	<0.05	^[15]^

DM = diabetes mellitus, EC = endometrial cancer, T2DM = type-2 diabetes mellitus, T1DM = type-1 diabetes mellitus.

## 3. The biological mechanisms linking DM and EC

Increasing evidence suggests that DM is closely related to an increased risk of EC, and the etiological relationship between DM and EC is complex and poorly understood. Although the precise biological mechanisms involved are not completely understood yet, the underlying mechanisms, including hyperglycemia, IR, hyperinsulinemia, glycolysis, chronic inflammation, obesity, and activation of specific signaling pathways, may contribute to the increased risk of EC in diabetic patients.

### 3.1. Hyperglycemia

Glucose is an essential nutrient for maintaining the cell energy balance. Evidence suggests that cancer cells are much more sensitive to glucose than different types of normal cells because of their higher energy expenditure.^[17]^ Glucose deficiency or low glucose concentration results in a kind of dilemma of energy crisis. It specifically or nonspecifically activates multiple death pathways that induce cell cycle arrest or apoptosis.^[18,19]^ A high glucose environment may promote tumor growth by providing EC cells with a carbon source, which can be used in a variety of biosynthetic pathways that are necessary to induce cell proliferation. In addition, the proteins and enzymes that regulate glucose metabolism have been proved to be viable therapeutic targets for EC.

A recent study using 2 EC cell lines revealed that low glucose suppressed cell growth and proliferation, induced prominent cell apoptosis, and led to cell cycle G1 arrest, while high glucose enhanced the adhesion and invasive abilities of EC cells, suggesting that glucose concentration is closely related to the growth of EC cells.^[8]^ Furthermore, hyperglycemia accounts for the induction of DNA damage and oxidative stress, thereby triggering the early stages of tumorigenesis.^[20]^ Based on the above, targeting glucose metabolism will be a promising strategy for EC therapy.

### 3.2. IR and hyperinsulinemia

Although EC is generally considered hormone-sensitive, its development is also profoundly affected by environmental and modern lifestyles. IR is an important factor leading to a variety of metabolic disorders, including prediabetes, T2DM, polycystic ovary syndrome (PCOS), and metabolic syndrome. IR is the main cause of T2DM and sometimes occurs before patients present clinical symptoms.^[21]^ This kind of prediabetic state plays a key role in the development and progression of several types of cancer, including colorectal cancer, prostate cancer, breast cancer, and EC.^[22]^ In particular, studies have shown that DM can increase the relative risk of EC, and the risk of EC also increases in nondiabetic women with IR.^[23]^ Moreover, further studies confirmed that patients with both IR and T2DM had a significantly increased risk of developing EC. As noted above, these studies indicate that IR is closely related to EC, and may reinforce each other.

Epidemiological data indicate that IR and its accompanying hyperinsulinemia are significant risk factors for EC and may also promote the development of EC.^[15,24]^ IR has been defined as a decrease in the efficiency of insulin to promote glucose uptake and utilization due to various reasons.^[25]^ In the early stage of IR, to maintain the stability of blood glucose levels, the pancreatic β-cells of the body compensatorily secrete excessive insulin, thereby inducing hyperinsulinemia. Accompanied by the failure of pancreatic β-cells, increased blood glucose levels ultimately lead to DM progression.^[26]^ Indeed, the risk of EC can decrease with improvement of IR and other metabolic abnormalities.^[27]^

At present, the mechanism of IR in EC development remains unclear, and some biological pathways may be involved. The direct effects of insulin and insulin-like growth factors (IGFs) on endometrial cells and changes in several important signaling pathways, including the phosphatidylinositol 3-kinase (P13K) and mitogen-activated protein kinase (MAPK)/extracellular signal-regulated kinase (ERK) pathways, may all play a key role in the occurrence and development of EC.^[28–30]^ Furthermore, studies have indicated that the binding of insulin to the vascular endothelial growth factor receptor (VEGFR) in endometrial cells promotes cell proliferation and inhibits apoptosis. However, it induces the expression of VEGF and promotes angiogenesis, which in turn leads to the occurrence of EC. In addition, hyperinsulinemia may also have an indirect effect on endometrial cells by increasing endogenous estrogen levels and reducing sex hormone-binding globulin (SHBG) levels.^[31]^ Under physiological conditions, 30–50% of the estrogen in plasma is present in an inactive form when combined with SHBG. However, IR and hyperinsulinemia in the body can inhibit the secretion of SHBG, thereby increasing free active estrogen in the blood circulation.^[32]^ At the same time, hyperinsulinemia can also increase the expression of estrogen receptor and in turn enhance the function of estrogen in the endometrium.^[33]^ As a result, excess insulin promotes the proliferation of endometrial cells under the action of estrogen, thereby increasing the risk of EC.

### 3.3. Glycolysis

The state of high glucose environment in diabetic patients may have various effects on the metabolism of EC cells. Glycolysis is a complex biological phenomenon that is mediated by various glycolytic genes and multiple glucose metabolism pathways, and the survival of EC cells is closely linked to glycolysis.^[34]^ The rapidly increase in glycolysis is a hallmark feature of various cancers, and the transport of glucose on the plasma membrane mediated by glucose transporters (GLUTs) is the first key step of glucose metabolism, which promotes the uptake of glucose by cancer cells and provides structural molecules needed for cancer progression.^[35]^ Studies have demonstrated that the expression of GLUTs with different affinities for glucose was increased in EC cells, among which GLUT1 is widely expressed in human tissues and participates in the absorption of glucose in an alkaline state,^[36]^ suggesting that EC cells may increase the utilization of glucose under various conditions via a variety of glucose transporters. At the same time, changes in the expression or activity of some proteins can also be observed in other experiments on EC cells, such as overexpression of epidermal growth factor receptor (EGFR), nuclear localization of β-catenin, liver kinase B1 (LKB1), deletion of tuberous sclerosis2 (TSC2), and mutant TP53. These factors stimulate glucose metabolism via various mechanisms. In addition to regulating GLUTs, glycolysis also increases the utilization of glucose by altering the activity of key glycolytic enzymes.^[37]^ It is worth noting that these key proteins in glycolysis also play an important role in insulin signal transduction, generally via the PTEN/PI3K/Akt/mTORC pathway, which contributes to glucose metabolism, cell growth, proliferation, survival, metastasis, and drug resistance in EC.^[38,39]^ Beyond that, studies have reported that high glucose increased glycolytic activity by activating both AMPK and AKT/ mTOR/ S6 pathways, and subsequently stimulated cell proliferation, while low glucose stimulated the expression of phosphorylated AMPK,^[8]^ suggesting that glucose may have direct or indirect effects on glycolysis, and AMPK can act as a switch involved in these signaling networks.

### 3.4. Chronic inflammation

Increasing evidence suggests that inflammation can trigger cancer development and progression. Inflammatory factors, such as tumor necrosis factor α (TNFα), interleukin-6 (IL-6), and cyclooxygenase-2 (COX-2), participate in carcinogenesis, which mediates cellular signal transduction, reduces tumor inhibitory function, increases cell cycling, and induces oncogene expression.^[40]^ In contrast, inhibition of inflammatory factors, such as NF-κB, can reduce the incidence of cancer. Notably, activation of the NF-κB signaling pathway plays a key role in certain types of cancers in women, such as EC, breast cancer, and ovarian cancer.^[41,42]^

In addition, NF-κB is also affected by the endometrial hormone environment, especially estrogen, which can activate the NF-κB signaling pathway and induce the expression of inflammatory cytokines, such as metalloproteinases (MMPs), TNFα, and interleukin-1 (IL-1), and in turn promote inflammatory processes.^[42]^ In patients with T2DM, IR and its accompanying hyperinsulinemia can trigger chronic inflammation, thereby aggravating insulin resistance.^[43]^ As a result, the above data have suggested that chronic inflammatory state in patients with T2DM may be a key molecular mechanism related to the occurrence and progression of EC.

### 3.5. Obesity

Obesity plays a pivotal role in linking DM and EC by acting as a joint confounder. The global prevalence of obesity was associated with an increased risk of DM and EC, and the summary RR for EC was 1.52.^[44]^ Besides, obesity has been demonstrated to be related to an overall increased risk of death and recurrence of cancer, among which there was a roughly 2-fold increase in EC mortality.^[45]^ There are multiple studies linking these 3 factors including adipokine pathways, hormonal control and inflammation.^[46]^ The excess adipose tissue acts as an endocrine organ, leading to hormone production, cellular proliferation, inflammatory responses, and IR.^[47]^ The excessive secretion of endogenous estrogen promotes the growth and proliferation of endometrial cells by activating both the estrogen receptor and IGF-1 receptor, which in turn activates the downstream PI3K-AKT-mTOR signaling pathway, thereby resulting in an increased risk of EC.^[48]^ In addition, the production and secretion of adipokines, including leptin, monocyte chemoattractant protein-1 (MCP-1), IL-6, IL-8, and TNF-α, are inextricably linked with carcinogenesis.^[47]^ In turn, IR ranks among one of the strongest risk factors for EC development. Taken together, these studies on weight management can provide new insights into obesity-associated EC.

### 3.6. Epithelial-mesenchymal transition (EMT)

EMT is a hallmark of tumorigenesis, invasion, and metastasis. There is a wealth of evidence that a high-glucose environment can induce EMT and therefore promote proliferation, invasion, and metastasis, but resist therapy in colon, breast, and pancreatic cancer cells.^[49,50]^ The loss of E-cadherin is often considered to be an important signal of EMT, which reduces the adhesion of cells and destroys the stability of epithelial structures. This process is accompanied by increased expression of mesenchymal-related proteins, including N-cadherin, vimentin, and fibronectin, which enhance the ability of invasion and metastasis of cancer cells.^[51]^ Studies on EC have shown that a high glucose environment enhances the adhesion and invasive capabilities of EC cells by reducing E–cadherin expression and increasing the expression of Snail and Slug, and the loss of E–cadherin was mainly caused by the overexpression of key transcriptional repressors Snail and Slug.^[8]^ In addition, changes in the expression of EMT-related markers are strongly associated with metastatic disease and decreased survival in cancers.^[52]^ Nevertheless, the specific molecular mechanisms by which DM promotes proliferation, invasion, and metastasis via EMT deserve further investigation.

## 4. Potential therapeutic strategies for EC

In recent years, the exploration of antidiabetic medications for the prevention and treatment of cancer has attracted increasing attention from researchers. Numerous studies have indicated that some antidiabetic medications are closely associated with the incidence of cancer.^[53]^ Commonly used antidiabetic agents include sulfonylureas, thiazolidinediones, diguanidine, and insulin analogs, each of which has a different mechanism of action in vivo. Certain types of sulfonylureas, thiazolidinediones, and insulin analogs have been shown to increase cancer risk, among which the long-acting insulin analog insulin Glargine could increase the risk of breast cancer,^[54,55]^ whereas metformin therapy significantly reduced the overall incidence and mortality rates of cancer by 14% and 30%, respectively,^[56,57]^ including EC and ovarian cancer. Another meta-analysis indicated that metformin therapy significantly reduced the overall risk of cancer by 27% in T2DM patients.^[58]^

Metformin is a first-line oral antihyperglycemic agent that is widely used to treat T2DM via improved insulin sensitivity. In addition, metformin also draws special attention to its anticancer effects and improves overall survival in the field of tumor therapy. Studies have shown that metformin plays a central role in inhibiting cell proliferation, invasion, and migration, inducing apoptosis, and promoting growth cycle arrest in EC.^[59]^ The possible mechanisms underlying the anticancer effects of metformin in EC have been studied. There is evidence that metformin can act directly on AMPK the TSC2/ mTOR/ S6 pathway and regulate cell growth and proliferation.^[60,61]^ When the concentration of metformin in vivo exceeds the physiological concentration, metformin can induce G0/G1 cell cycle arrest, apoptosis, autophagy, and increase AMPK phosphorylation.^[59]^ Additionally, recent findings have indicated that metformin treatment can inhibit the EMT process in EC cells by increasing E-cadherin expression and reducing the expression of mesenchymal markers such as N-cadherin, vimentin, and fibronectin, along with EMT transcriptional factors including Snail-1, Twist-1, and ZEB-1,^[62,63]^ emphasizing its biological effects on EMT and the consequent clinical significance for EC therapy.

A randomized clinical trial study revealed that metformin induced endometrial atrophy or restored endometrial histology in 95.5% of patients with EC, while the megestrol treatment group had a prevalence of 61.9% according to statistics. It is worth noting that 2 low-grade EC patients at stage Ia developed atrophic endometrium after receiving metformin treatment for 3 months in this study,^[64]^ suggesting that metformin can act as an effective antiestrogenic drug for controlling abnormal endometrial hyperplasia. Moreover, since young women suffering from PCOS have a high risk of EC, another study found that Diane-35 combined with metformin treatments had the potential to transform EC into normal endometrium in young female patients with early stage EC and IR.^[65]^ However, the underlying mechanisms behind this effect require further research. Furthermore, studies have revealed that the expression of estrogen receptors as well as hyperinsulinemia-associated molecules including insulin, glucose, leptin, and IGF-1 was significantly decreased in EC patients after metformin therapy,^[66–68]^ indicating an anti-proliferative effect on EC following treatment with metformin.

Taken together, the current evidence suggests that metformin may provide a new perspective for physicians in the treatment of diabetic patients with EC. The above findings may provide new insights into the prevention and treatment of malignant endometrial tumors, especially for young patients who desperately need to preserve fertility potential in the early stages of EC.

## 5. Conclusions

**Figure 1.**
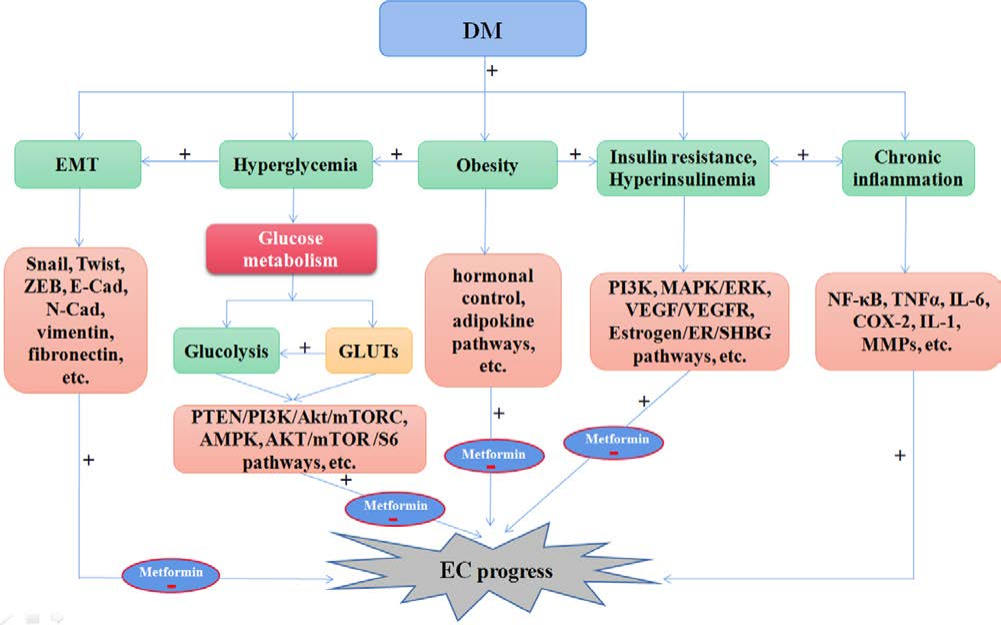
Schematic diagram of the effects of DM on EC regarding hyperglycemia, insulin resistance, hyperinsulinemia, glycolysis, chronic inflammation, obesity, activation of signaling pathways and pharmacological therapy for EC associated with DM. Green boxes indicate possible biological mechanisms linking DM and EC, and pink boxes indicate related signaling pathways involved. “+” represents positive regulation, and “−” represents negative regulation. DM = diabetes mellitus, EC = endometrial cancer, EMT = epithelial-mesenchymal transition.

**Figure 2.**
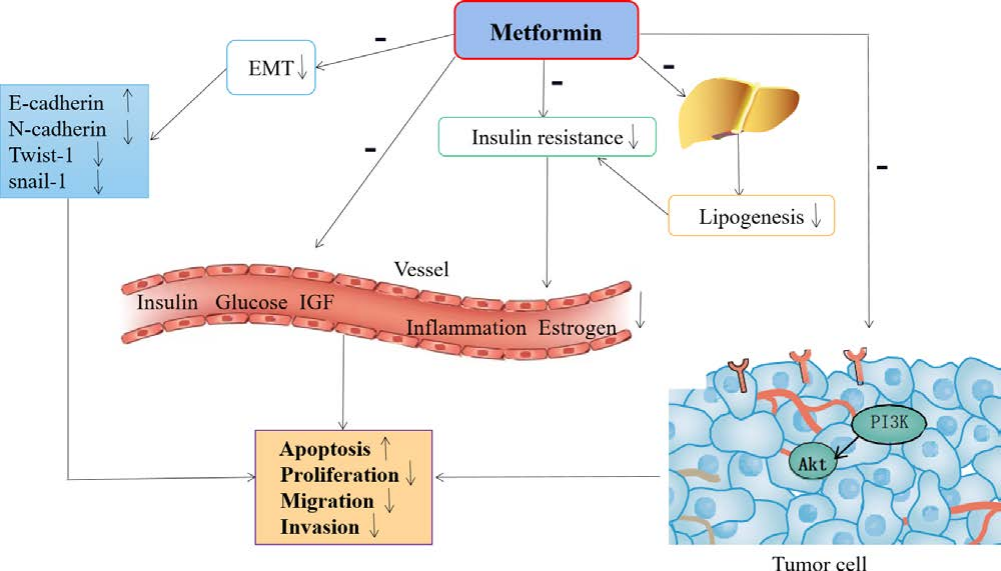
Mechanisms of metformin in inhibiting cell proliferation, invasion, migration, and inducing apoptosis in EC regarding EMT, inflammation, insulin resistance, lipogenesis and so on. “+” represents positive regulation, and “−” represents negative regulation. EC = endometrial cancer, EMT = epithelial-mesenchymal transition.

At present, the prevalence of DM has been increasing, resulting in an increased risk of EC, and the onset age of EC tends to be younger. For patients with early stage of EC, there is a desperate need to preserve fertility potential. In addition, there is still a lack of effective treatments for patients with advanced EC, thus, further investigations into preventive and therapeutic strategies for EC are necessary. Increasing evidence suggests that DM is closely related to an increased risk of EC, and the etiological relationship between DM and EC is complex and poorly understood. In this review, we provide an overview of the effects of DM on EC in hyperglycemia, IR, hyperinsulinemia, glycolysis, chronic inflammation, obesity, activation of signaling pathways involved, and pharmacological therapy of EC associated with DM (Fig. 1). Some DM drugs, such as metformin, can exert a beneficial effect on EC therapy due to its potential to reduce recurrence and occurrence, and improve the overall survival through several ways, including reducing the biological effects of the EMT process (Fig. 2). Early treatment with metformin is expected to be an effective adjuvant treatment for EC in the future.

In addition, the therapeutic targets and prognosis of EC tend to differ depending on the histological type and molecular pathological classification. EC can be divided into 2 histological types according to its pathogenesis: type I estrogen-dependent EC and type II estrogen-nondependent EC.^[69]^ Type I EC accounts for 80–90% of the sum, and the main histological type is adenocarcinoma. This type of patient is younger and closely related to diabetes, obesity, hypertension, infertility, and postmenopausal delay. Moreover, it is usually PR-positive, and the majority of cases have a better prognosis.^[70]^ However, type II EC accounts for only a small proportion, and its occurrence is not very close to endocrine and metabolic factors, including estrogen and DM. The histological types of type II EC are mainly serous carcinoma and clear cell carcinoma, and type II EC is usually PR-negative, with high malignancy and poor prognosis.^[71]^ In terms of molecular changes, there are also differences between the 2 types: type I EC is mainly related to PTEN, KRAS, CTNNB1, and PIK3CA mutations and microsatellite instability (MSI), whereas type II EC is mostly associated with HER2 amplification, P53 mutation, and inactivation of E-cadherin.^[72]^ In general, according to current evidence, endocrine and metabolic factors, including DM, may be more closely related to type I EC. On the other hand, some studies have shown that the 2 types of EC have multiple similar risk factors, and thus, there may be no obvious boundary between them. As a result, 1 limitation existed in this study was that the deeper relationship between DM and histopathological and molecular pathological subtypes of EC was insufficient. Further investigations are necessary to focus on the molecular phenotypes and histological subtypes of endometrial tumors and elucidate the different etiological relationships between DM and EC, and the specific effects of antidiabetic medicines on the risk of EC.

In conclusion, this study updated and further improved the most recent evidence regarding the etiological relationship between DM and EC, provided a global view of the effects of DM on EC regarding hyperglycemia, insulin resistance, hyperinsulinemia, glycolysis, chronic inflammation, obesity, and activation of signaling pathways involved, and reinforced the notion of clinical application of antidiabetic medications for EC with suggestions. In addition, we also focused on the relationship between DM and histopathological and molecular phenotypes of EC to broaden the perspective regarding the different etiological relationships. This knowledge has significant value for further opening up more preventive and therapeutic strategies for EC by targeting glucose metabolism.

## Acknowledgments

The authors gratefully acknowledge the Natural Science Foundation of Hubei Province for the funding support (grant no. 2019CFB274).
